# Preparation of One-Dimensional Polyaniline Nanotubes as Anticorrosion Coatings

**DOI:** 10.3390/ma15093192

**Published:** 2022-04-28

**Authors:** Guangyuan Yang, Fuwei Liu, Ning Hou, Sanwen Peng, Chunqing He, Pengfei Fang

**Affiliations:** 1 China Tobacco Hubei Industrial Cigarette Materials, LLC, Wuhan 430051, China; yangguangyuan@whut.edu.cn (G.Y.); houning@hjl.hbtobacco.cn (N.H.); 2 Key Laboratory of Artificial Micro- and Nano-Structures of Ministry of Education, Department of Physics, Wuhan University, Wuhan 430072, China; liufuwei168@163.com (F.L.); hecq@whu.edu.cn (C.H.)

**Keywords:** polyaniline, nanotubes, one-dimensional, electrochemical performance

## Abstract

Uniform polyaniline (PANI) nanotubes were synthesized by a self-assembly method under relatively dilute hydrochloric acid (HCl) solution. Scanning electron microscopy (SEM), Fourier transform infrared (FTIR) spectroscopy, and UV-Vis-NIR spectroscopy were employed to characterize the morphology and molecular structure of the PANI products. SEM images show that the PANI nanotubes have uniform morphology and form compact coating on the substrate surface. For comparison, aggregated PANI was also synthesized by conventional polymerization method. The performance of the PANI products on carbon steel was studied using eletrochemical measurement and immersion corrosion experiment in 3.5 wt% NaCl aqueous solution. The corrosion potentials of carbon steel samples increase by 0.196 V and 0.060 V after coated with PANI nanotubes and aggregated PANI, respectively, and the corrosion currents density decrease by about 76.32% and 36.64%, respectively. The 6-day immersion experiment showed that the carbon steel samples coated by PANI nanotubes showed more excellent anticorrosion performance, because the more compact coating formed by PANI nanotubes may inhibit the corrosion process between the anodic and cathodic.

## 1. Introduction

Conducting polymers have been studied for their excellent protective ability for metals in the last few years. Polyaniline is generally considered to be one of the best candidates for an excellent anticorrosion film, because of its ease of synthesis [1,2,3,4,5], interesting redox properties [6,7], and good environment stability [8,9]. Although enormous efforts have been devoted to the study of electrochemical polymerization [10,11,12,13], it is not easy to make large polyaniline films in this method and some corrosion-susceptive metals are often oxidized or dissolved in the potential domain of the polyaniline electro-polymerization. Another strategy explored was the use of the chemical polymerization method. However, most of the products in conventional polymerization method are aggregated PANI with poor solubility in conventional solvents, which is unfavorable to the formation of compact films.

In order to obtain large-area and compact coatings, specific PANI nanostructures synthesized by chemical polymerization may be an effective method to improve the solubility and dispersion stability [14,15,16]. It is generally considered that the compact PANI film itself can generate compact passive layers on the metal surface and inhibit the corrosion process between anodic and cathodic [17,18,19,20]. Therefore, some researchers have tried to explore novel PANI nanostructures, especially one-dimension nanostructures, for anticorrosion application. Yao et al. [15] synthesized polyaniline nanofibers with good solubility and dispersion stability in ethanol, and applied them to carbon steel surfaces as a suspension in ethanol and investigated the anticorrosion performance. The results showed that PANI nanofibers bring a better passive effect to carbon steel and provide more excellent corrosion protection than aggregated PANI. Yang et al. [16] synthesized polyaniline nanostructures in sulfuric acid solution using three different polymerization methods. They found that nanofibers synthesized by the direct mixed reaction method have highly uniform morphology which may improve the solubility and the formation of the compact coating thus effectively enhancing the corrosion protection property. The polyaniline nanotube is another one-dimension nanostructure which has attracted a great deal of attention because of its potentially interesting electrical and optical properties [21,22,23]. Moreover, PANI nanotubes with uniform morphology may have good solubility and dispersion stability which is necessary in the formation of compact coatings. On the other hand, it was found that pure PANI coating film had a good protective effect, but its corrosion protection was short term due to its poor barrier to water. It is necessary to develop a blended coating containing both conventional resin and PANI [19]. Although there are several previous reports of PANI nanotubes used as anticorrosion coating, the preparation of PANI nanotubes and design of high-performance anticorrosion PANI nanotube/polymer coatings are still a great challenge.

In this study, polyaniline nanotubes were prepared under a relatively dilute HCl solution. The obtained polyaniline products were not only directly applied on carbon steel surface but also as fillers dispersed in alkyd resin to investigate their anticorrosion performance. The results show that PANI nanotubes have more excellent protective properties than aggregated PANI. The protection mechanism was also discussed.

## 2. Materials and Methods

### 2.1. Materials

Aniline (analytical grade) was obtained from Tianjin Tianli Chemical Reagent Co. Ltd. and purified by distillation prior to use. Ammonium persulfate (APS), N-methyl-2-pyrrolidone (NMP), hydrochloric acid, and methanol were analytical grade and used without further purification. The alkyd resin we used was 3139 alkyd resin of Hubei Wuhan Lion Rock brand (viscosity: ≥300/25 °C, solid: 65 ± 2%, acid value: ≤10 mgkoh/g). All aqueous solutions were prepared with deionized water by an ultrapure water treatment system.

### 2.2. Preparations

PANI nanotubes were prepared by a self-assembly method under relatively dilute HCl solution. One milliliter of aniline was dissolved in 10 mL of 1 mol/L HCl and 2.5 g APS was dissolved in 40 mL deionized water. The two solutions were then mixed and immediately shaken well for approximately 5 min. The reaction was carried out at room temperature for an extra 24 h. The reaction products were washed with deionized water and centrifuged to separate PANI. Separated PANI was then dispersed in deionized water and centrifuged. This treatment was repeated several times until the suspension reached a neutral pH and became colorless. The resulting polyaniline precipitate was centrifuged and repeatedly washed using methanol to remove the oligomer and finally dried in an oven at about 60 °C for 24 h.

In the conventional polymerization method, 1 mL of aniline was added to 10 mL of 1 mol/L HCl solution, and the mixed solution was transferred to an ice bath environment. Then 40 mL pre-cooled aqueous solution of APS (0.25 mol/L) was added dropwise to the pre-cooled aniline-acid mixed solution with constant stirring. The reaction was conducted at 5 ± 1 °C. After the addition, the stirring was continued for 1 h for ensuring complete polymerization. Then the reaction products were purified using deionized water and methanol. The operation is similar to the subsequent treatment of PANI nanotubes.

The rectangular (28 mm × 24 mm × 1 mm) carbon steel (the content of carbon is 0.22–0.45%) samples were polished by emery paper 1000 grit, and all steel working samples were treated in acetone and ethanol solution to degrease prior to coating. A certain quality of aggregated PANI or PANI nanotubes was dissolved in NMP to obtain a 10% suspension respectively.

The obtained PANI products were also used for the preparation of the aggregated PANI/alkyd and PANI nanotube/alkyd coatings. The coating systems were prepared separately by dispersing 2.0, 5.0, and 8.0 wt% of polymers (aggregated PANI and PANI nanotubes) in 20 wt% solution of alkyd in xylene. Additionally, the alkyd without PANI was prepared as well.

Both of the PANI and PANI/alkyd coatings were allowed to dry in air at room temperature for 144 h, then the coated samples were encapsulated by paraffin and a constant area of about 1 cm^2^ was left for corrosion test.

### 2.3. Characterization

**Figure 1:**
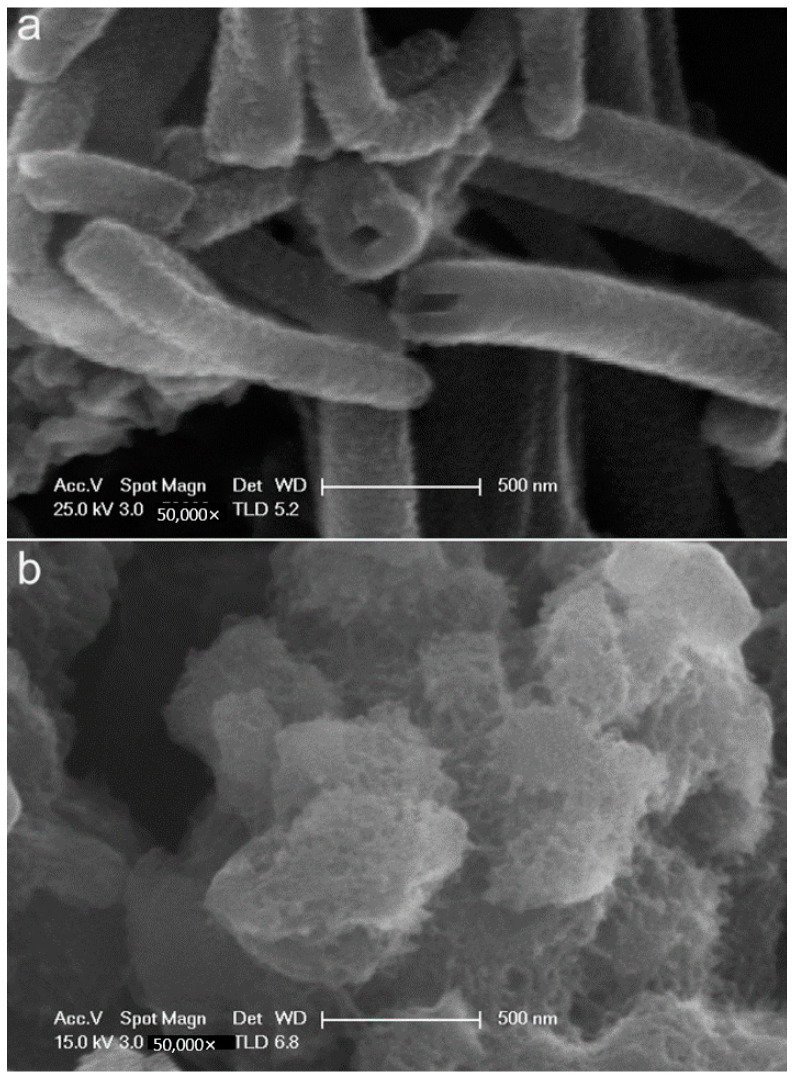
SEM images of PANI nanotubes (**a**), and aggregated (**b**).

**Figure 2:**
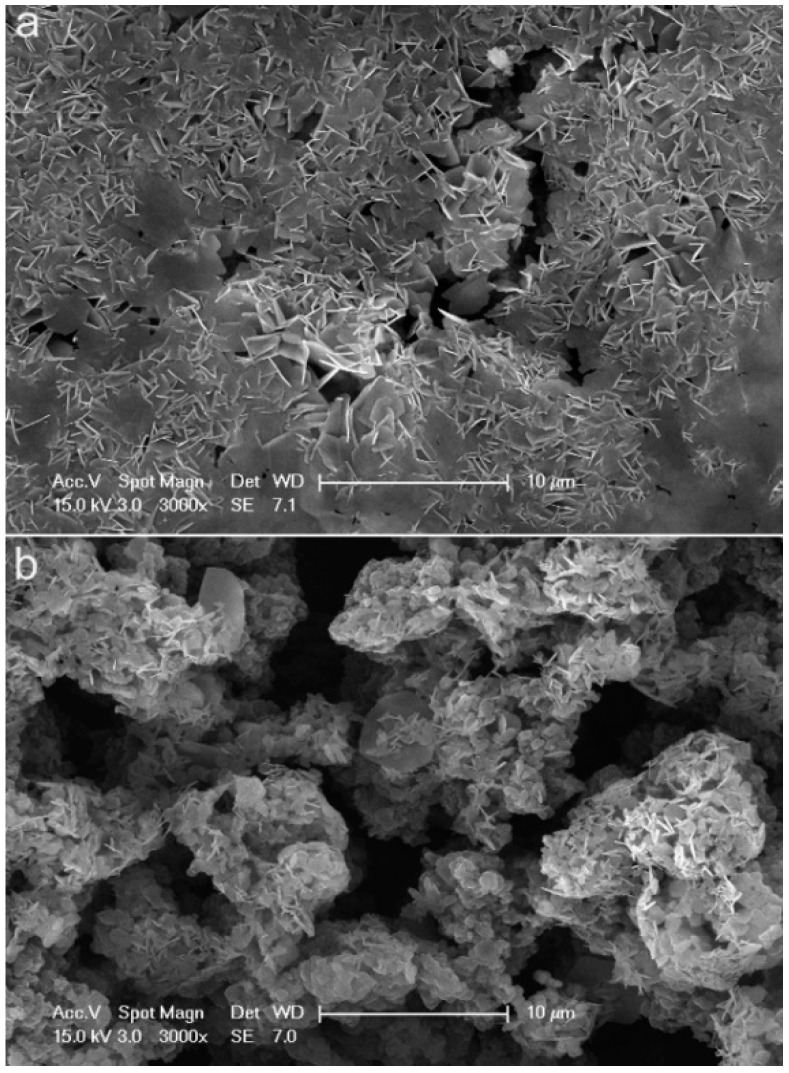
SEM images of the coatings of PANI nanotubes (**a**), and aggregated PANI (**b**).

The thicknesses of the PANI alone and PANI/alkyd coatings were measured using a magnetic thickness gauge (QCC-A, Jiangdu Pearl Experimental Machinery Factory, Yangzhou, China). Morphologies of the PANI products and the coatings were investigated by a Sirion field-emission scanning electron microscopy (Hitachi S-4800, FEI Company, Hillsboro, OR, USA). The magnifications of PANI nanotubes and aggregated PANI in SEM (Figure 1) are both 50,000, and the magnifications of the PANI nanotubes and aggregated PANI coatings (Figure 2) are both 3000. The molecular structures of polyaniline nanostructure were studied by an attenuated total reflectance Fourier transform infrared spectrometer (ATR-FTIR, Nicolet iS10, Thermo Electron Corporation, Waltham, MA, USA) and a Cary 5000 UV-Vis-NIR spectrometer (Agilent Technologies, Ltd, Santa Clara, CA, USA). In ATR the frequency range was from 550 to 4000 cm^−1^, number of scans was 32, and the resolution used was 6 with dataspacing 1.929 cm^−1^. The electrochemical corrosion measurements were performed on a potentiostat/galvanostat (CHI 600C, Chenhua Company, Shanghai, China) in a three-electrode electrochemical cell using carbon steel samples as working electrodes. A Pt sheet was used as a counter electrode and all potentials were referred to the saturated calomel electrode (SCE). In Tafel plot, the scanning rate was 10 mV/s. The electrochemical impedance spectroscopy (EIS) measurements were taken in the frequency range of 100 k to 0.01 Hz, and the amplitude of the sinusoidal voltage signal was 10 mV. The EIS data were analyzed and fitted with ZSimpWin software. In both Tafel and EIS experiments, the corrosion environment was 3.5 wt% NaCl water solutions at room temperature.

## 3. Results and Discussion

Figure 1 shows the SEM images of PANI nanotubes and aggregated PANI. It is found that the PANI nanotubes have nearly uniform outer diameters of 180–250 nm, wall thicknesses of 40–80 nm, and lengths varying from 500 to 2000 nm (Figure 1a). However, the PANI synthesized by conventional polymerization method are irregularly shaped agglomerates containing varies of particulates (Figure 1b). In our experiments it is evident that a relatively dilute acidic condition was obtained when the APS solution was mixed with the aniline–acid solution directly, while a higher acidity was kept when the APS solution was added dropwise. The PANI nanotubes are proposed to produce within an intermediate acidity interval, and granular PANI are obtained at higher acidity [24]. Polymerization starting in mildly acidic conditions results in aniline oligomers, which are insoluble in water [25,26]. These aniline oligomers may be made of phenazine-like moieties oxidized from ortho-coupled aniline. They aggregate to constitute a template-like structure, which further dictates the directional growth of PANI, namely the production of PANI nanotubes. In addition, the final nanotubular productions are generated through the self-organized phenazine units and the stacking of aniline oligomers [24,25,27,28,29,30,31,32].

The thicknesses of aggregated PANI and PANI nanotubes coatings were measured at 8 ± 0.3 μm. The SEM morphologies of polyaniline coatings formed using PANI nanotubes and aggregated PANI are presented in Figure 2. It can be seen that the coating (Figure 2b) formed by aggregated PANI shows agglomerates and some cracks on the coating. In the presence of PANI nanotubes, the image (Figure 2a) gave the formation of a more uniform and compact coating. These different macroscopic properties of the polyaniline coatings could directly affect anticorrosion performance.

**Figure 3:**
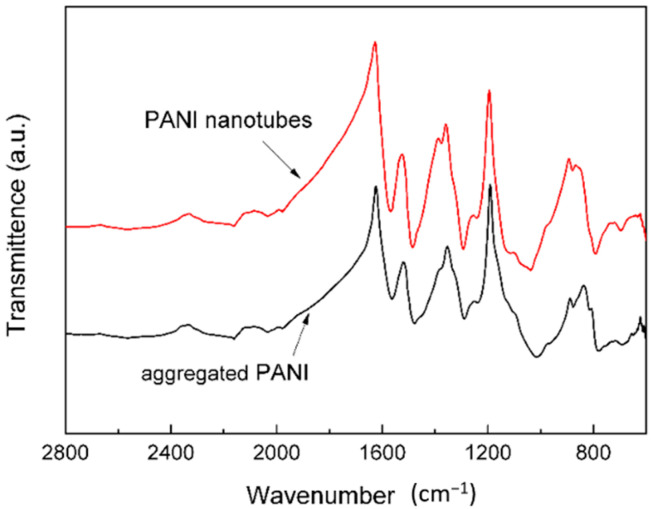
FT−IR spectra of PANI nanotubes and aggregated PANI.

**Figure 4:**
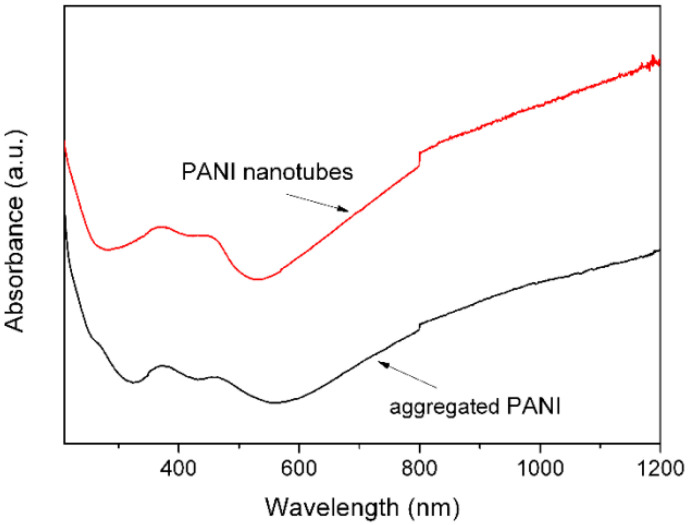
UV-Vis-NIR spectra of PANI nanotubes and aggregated PANI.

The FTIR spectra of the PANI nanotubes and aggregated PANI are given in Figure 3. The PANI nanotubes and aggregated PANI have similar FTIR spectra. The peaks at 1570 and 1487 cm^−1^ are the stretching mode of C=N and C=C for the quinoid and benzenoid rings. The peaks at 1294 and 1244 cm^−1^ are assigned to the C−N stretching mode of benzenoid ring, while the peaks at 1130 and 800 cm^−1^ are attributed to the aromatic C−H in-plane bending and the out-of-plane deformation of C−H in the 1,4-disubstituted benzene ring, respectively [33]. These values are similar to the previously reported infrared spectra for polyaniline systems [34,35]. Figure 4 shows the UV-Vis-NIR spectra of the polyaniline structures. Two distinctive absorption bands at 374.5 and 468 nm can be seen in the UV-Vis-NIR spectra. The former is associated with the π → π* transition and the latter is caused by polaron band → π* transition [33,36]. FTIR and UV-Vis-NIR results show that the states of the nanostructures should be doped polyaniline in their emeraldine salt forms [16].

**Figure 5:**
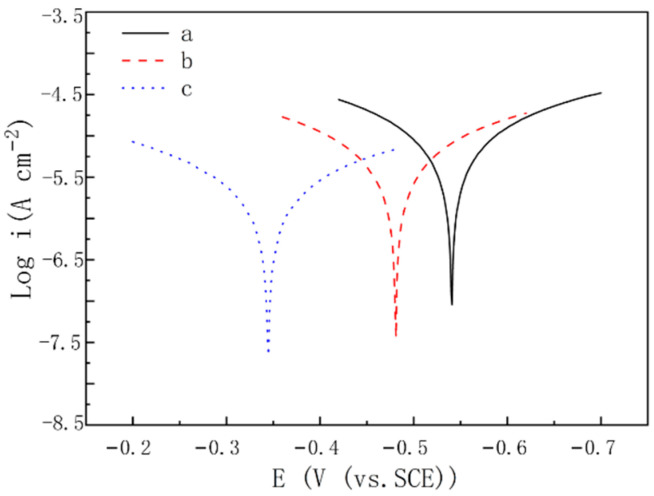
Tafel curves for uncoated CS (a), CS−A−PANI (b), and CS−N−PANI (c) in 3.5 wt% NaCl aqueous solution.

The potentiodynamic polarization curves for uncoated carbon steel (CS), carbon steel covered with aggregated PANI (CS-A-PANI), and carbon steel covered with PANI nanotubes (CS-N-PANI) in 3.5 wt% NaCl solution are shown in Figure 5. The values of corrosion potential (*E*_corr_) and corrosion currents density (*I*_corr_) obtained from the potentiodynamic polarization curves are presented in Table 1. Compared with uncoated carbon steel, the *E*_corr_ of CS-A-PANI and CS-N-PANI increased about 0.060 V and 0.196 V, while the *I*_corr_ of CS-A-PANI and CS-N-PANI decreased about 36.64% and 76.32%, respectively. When the carbon steel covered with aggregated PANI and nanotube PANI, the value of cathodic Tafel constants decrease from 95.1 mV/dec to 85.3 and 71 mV/dec, simultaneously the value of anodic Tafel constants decrease from 85.5 mV/dec to 70.9 and 66.5 mV/dec. These results indicate that PANI nanotubes have better corrosion protection to carbon steel than aggregated PANI. This is possibly due to PANI nanotubes possess favorable adhesion to carbon steel and guarantee the generation of the passive layer on the carbon steel surface [37].

**Figure 6:**
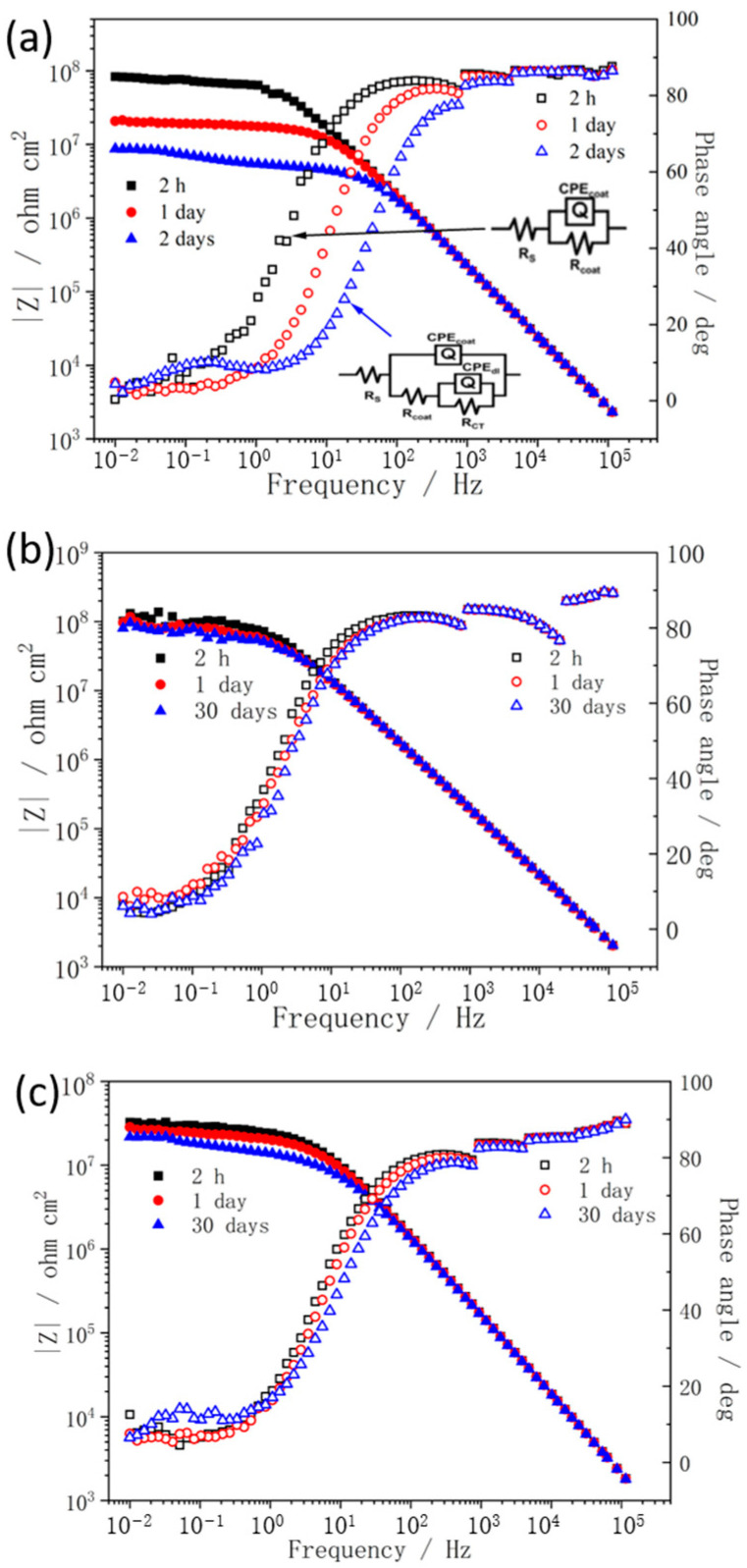
Impedance spectra for carbon steel coated with Alkyd (**a**), Alkyd−A2 (**b**), and Alkyd−A5 (**c**), in 3.5 wt% NaCl solution.

To further study the corrosion protection performance of PANI nanostructures, impedance spectra were employed to investigate the anticorrosion properties of the obtained PANI/alkyd coating system during immersion in 3.5 wt% NaCl solution. Figure 6 shows the impedance spectra of the alkyd coating containing aggregated PANI (the filler content is 0, 2, and 5 wt% and the obtained samples are abbreviated as Alkyd, Alkyd−A2, and Alkyd−A5, respectively). The obtained EIS spectra were fitted by two different equivalent circuits (depicted in Figure 6), in which R_s_, R_c_, and R_ct_ are the electrolyte solution resistance, coating resistance, and charge transfer resistance, respectively, while Q_c_ and Q_dl_ represent constant phase elements (CPE) associated with coating capacitance and double layer capacitance, respectively. As can be seen from Figure 6a, the impedance modulus of pure alkyd coating decreased with immersion time. After only two days of immersion, the impedance modulus at 0.01 Hz became less than 10^7^ Ω·cm^2^. Meanwhile, the second time constant can be also observed, which indicates that the electrochemical reactions at coating/metal interface took place. For the coating incorporated with PANI, it can be clearly seen (Figure 6b) that the addition of a small amount (2 wt%) of aggregated PANI significantly enhanced the anticorrosion properties of the alkyd coating, after 30-day immersion the impedance modulus also remains nearly 10^8^ Ω·cm^2^. However, with increasing aggregated PANI content (5 wt%), a dramatic decrease in impedance modulus is observed (Figure 6c). After 30 days of immersion, the time constant associated with the corrosion reactions at coating/metal interface also appears. Generally, the pure alkyd coating sample is partially heterogeneous and has lots of micro-defects, which affect the barrier properties of the polymer coating. The addition of a small amount of aggregated PANI may block the defects in some extent and thus form more compact coating structure, whereas an excess of aggregated PANI may agglomerate and affect the homogeneity of the coating, which result in the generation of some new micro-paths for corrosive media transportation [19].

**Figure 7:**
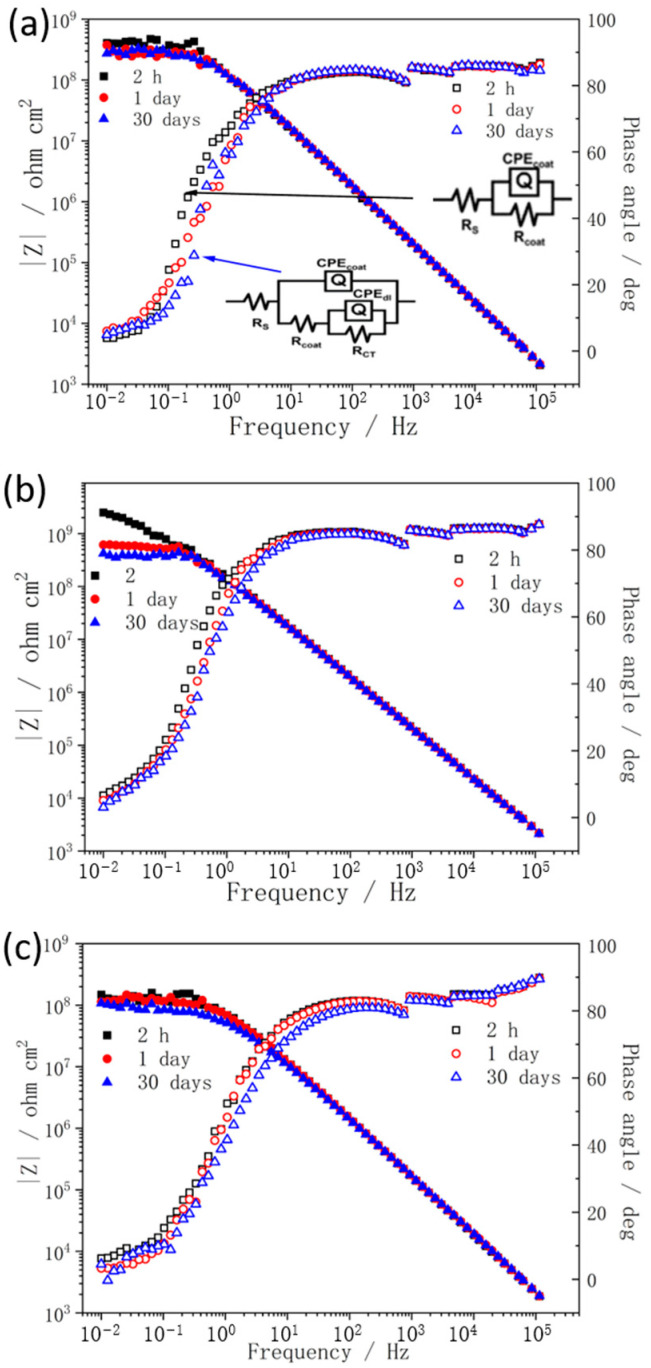
Impedance spectra for carbon steel coated with Alkyd−T2 (**a**), Alkyd−T5 (**b**), and Alkyd−T8 (**c**), in 3.5 wt% NaCl solution.

For PANI nanotube/alkyd coating system (the samples containing 2, 5, and 8 wt% of PANI nanotubes are abbreviated as Alkyd−T2, Alkyd−T5, and Alkyd−T8, respectively), the variation of impedance spectra (as shown in Figure 7) shows a similar trend with that of aggregated PANI/alkyd coatings. The difference is that with the increase in nanotube content, the anticorrosion property does not decrease until the filler content is higher than 5 wt%. Additionally, after 30-day immersion, all the PANI nanotube/alkyd coatings remain relatively high impedance modulus, more than 10^8^ Ω·cm^2^. The values of Rc and Qc were also evaluated using the fits of experimental spectra and are shown in Table 2. Comparing the obtained data, it can be clearly observed that values of R_c_ and Q_c_ increase and decrease at first and then decrease and increase with the increase in filler (aggregated PANI or PANI nanotubes) content, respectively. The optimum content of aggregated PANI and PANI nanotubes are 2 wt% and 5 wt%, respectively. All these results are in agreement with those obtained from the impedance modulus, which indicates that PANI nanotubes are easier to disperse in alkyd resin forming more compact structures, and thus show more excellent protective properties than the aggregated PANI.

**Figure 8:**
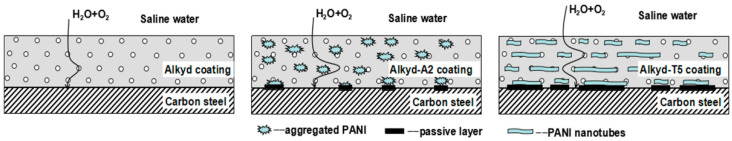
Mechanism of corrosion protection of alkyd containing PANI nanostructures.

Based on above discussion, the protection mechanism of PANI nanotubes was schematically illustrated in Figure 8. It was widely reported that PANI prevents corrosion of steel in two ways [27,33,35]. On the one hand, it acts as a physical barrier preventing penetration of corrosive media across the film. On the other hand, it induces the formation of passive layer on the steel surface. As can be clearly seen, the PANI nanotubes with uniform diameters can be easily dispersed in alkyd resin, forming a more compact coating structure, and thus preventing the corrosive ions transport through the coating. In addition, the relatively high PANI nanotube content in the alkyd coating doubtlessly ensures the good physical contact between PANI and steel substrate, facilitating the formation of passive layer. All these factors guarantee the excellent protection properties of PANI nanotubes.

## 4. Conclusions

Polyaniline nanotubes with uniform morphology were synthesized under relatively dilute acidic conditions. Phenazine-like moieties generated in mildly acidic conditions dictated the growth of PANI nanotubes. The obtained PANI products were not only directly applied on the carbon steel but also dispersed in alkyd resin as fillers to investigate their anticorrosion properties in the corrosive saline media. The results show that PANI nanotubes have more excellent protection properties than the aggregated PANI obtained by conventional polymerization method. The excellent anticorrosion properties of PANI nanotubes may be due to the formation of the compact coating on the carbon steel surface, guaranteeing the generation of the passive layer on the substrate surface. Moreover, a sufficient amount of PANI nanotubes ensures the homogeneity of the alkyd coating improving the barrier properties of the polymer film, as well as a good physical contact between PANI and steel substrate facilitating the formation of the passive layer.

## Figures and Tables

**Table 1:** Tafel curves parameters for uncoated CS, CS-A-PANI, and CS-N-PANI in 3.5 wt% NaCl solution.

Electrodes	*I*_corr_ (μA cm^−2^)	*E*_corr_ (V)	*b_a_* (mV/dec)	*b_c_* (mV/dec)
Uncoated CS	9.514	−0.541	85.5	95.1
CS-A-PANI	6.028	−0.481	70.9	85.3
CS-N-PANI	2.253	−0.345	66.5	71

**Table 2:** Electrochemical parameters calculated from EIS spectra of alkyd coatings containing PANI nanostructures immersed in 3.5 wt% NaCl solution.

Samples	Rc (Ω · cm^2^)		CPE_c_−T (F · cm^−2^)		CPE_c_−P	
	Value	Error (%)	Value	Error (%)	Value	Error (%)
Alkyd	1.48 × 10^7^	1.54	2.55 × 10^−9^	5.45	0.90	0.62
Alkyd−A2	7.90 × 10^7^	5.31	1.69 × 10^−9^	2.01	0.92	0.26
Alkyd−A5	3.01 × 10^7^	2.88	2.43 × 10^−9^	5.54	0.90	0.69
Alkyd−T2	7.38 × 10^8^	9.14	1.26 × 10^−9^	5.89	0.93	0.79
Alkyd−T5	1.32 × 10^9^	4.87	1.08 × 10^−9^	4.04	0.95	0.55
Alkyd−T8	5.30 × 10^7^	1.42	2.31 × 10^−9^	1.96	0.91	0.26

## Data Availability

Raw data is available upon request.
